# Mood and microbes: a comprehensive review of intestinal microbiota’s impact on depression

**DOI:** 10.3389/fpsyt.2024.1295766

**Published:** 2024-02-09

**Authors:** Ameer Luqman, Mei He, Adil Hassan, Mehtab Ullah, Liyuan Zhang, Muhammad Rashid Khan, Ahmad Ud Din, Kamran Ullah, Wei Wang, Guixue Wang

**Affiliations:** ^1^ Key Laboratory for Biorheological Science and Technology of Ministry of Education, National and Local Joint Engineering Laboratory for Vascular Implant, Bioengineering College of Chongqing University, Chongqing, China; ^2^ Chongqing University Cancer Hospital, Chongqing, China; ^3^ Chongqing Key Laboratory of Nano/Micro Composite Materials and Devices, Chongqing University of Science and Technology, Chongqing, China; ^4^ JinFeng Laboratory, Chongqing, China; ^5^ Plants for Human Health Institute, Department of Food, Bioprocessing and Nutrition Sciences, North Carolina State University, Kannapolis, NC, United States; ^6^ Department of Biology, The University of Haripur, Haripur, Pakistan

**Keywords:** depressive disorder, gut microbiota, gut-brain axis (GBA), dysbiosis, metabolites, probiotics, prebiotics, FMT

## Abstract

Depression is considered a multifaceted and intricate mental disorder of growing concern due to its significant impact on global health issues. The human gut microbiota, also known as the “second brain,” has an important role in the CNS by regulating it through chemical, immunological, hormonal, and neurological processes. Various studies have found a significant bidirectional link between the brain and the gut, emphasizing the onset of depression therapies. The biological and molecular processes underlying depression and microbiota are required, as the bidirectional association may represent a novel study. However, profound insights into the stratification and diversity of the gut microbiota are still uncommon. This article investigates the emerging evidence of a bacterial relationship between the gut and the brain’s neurological system and its potential pathogenicity and relevance. The interplay of microbiota, immune system, nervous system neurotransmitter synthesis, and neuroplasticity transitions is also widely studied. The consequences of stress, dietary fibers, probiotics, prebiotics, and antibiotics on the GB axis are being studied. Multiple studies revealed the processes underlying this axis and led to the development of effective microbiota-based drugs for both prevention and treatment. Therefore, the results support the hypothesis that gut microbiota influences depression and provide a promising area of research for an improved knowledge of the etiology of the disease and future therapies.

## 1 Introduction

Depression represents a frequent psychiatric disorder hampered by social stigma, a lack of efficient therapies, and a lack of adequate mental health facilities. It is characterized by persistent poor mood and lasts for at least two weeks formally defined as depression (1–3). Nearly 280 million human beings globally (21%) suffer from depression, although this disorder is commonly misdiagnosed and untreated for a variety of reasons (4). Numerous internal and environmental variables contribute to major depression, which affects millions of people every year (5). It is believed that the 400-1000 bacterial species that live in the gut of humans affect the brain’s nervous system. The human gut which is populated by 10^14^ microbes ten times more than human cells is an important part of the microbiota ecology (6). A growing amount of interest is being placed on the beneficial relationship between intestinal bacteria and their host that affects cell hormone release, which has an impact on host metabolism and the development of metabolic diseases (7, 8). In developed nations, about one out of every five individuals has experienced a depressive disorder at some point in their lives. Moreover, depressive conditions tend to be more distinct in nearly one-third of economically poor nations (9, 10).

**Figure 1:**
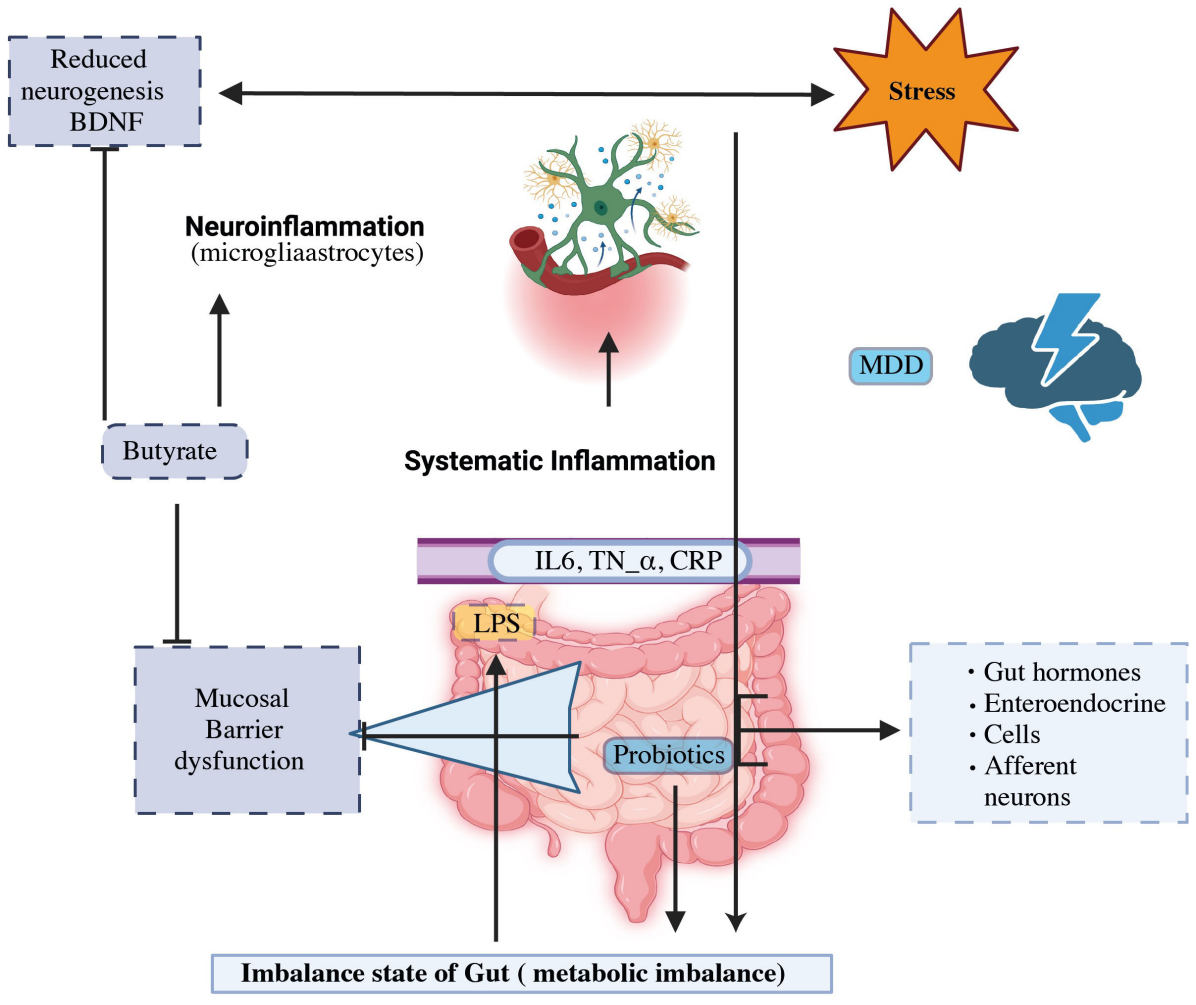
The gut-brain axis and probiotics’ potential to modulate MDD mechanism.

Although the origins of the disease are still unknown, several concepts have been put out to explain the etiology of its underlying mechanisms. Selective serotonin reuptake inhibitors (SSRIs) and serotonin-noradrenaline reuptake inhibitors (SNRIs) are extensively employed as antidepressants. These medications were developed by the monoamine hypothesis, which may be attributed to a deficiency in monoamine neurotransmitters (4, 11). In 2016, the World Health Organization (WHO) provided an estimate of 785,000 suicides taking place annually, resulting in a suicide rate of 10.6 suicides per 100,000 individuals (12). Notably, significant depression may be present in up to 60% of these instances (11). It may be able to expand the range of treatments and preventive therapies for patients suffering from major depression disorder (MDD) by better understanding the role of the gut microbes and the interaction between antidepressants and the axis of the gut-brain. Recent breakthroughs in MDD treatment emphasize the importance of intestinal microbes as a study domain (13, 14). Based on evidence from twin, family, and epidemiological studies, environmental factors and their interplay with hereditary factors have a significant impact on MDD (15, 16). **Figure 1** illustrates the efficacy of probiotics, particularly butyrate-producing bacteria, to alleviate the physiological causes of MDD while also structurally modulating the intestinal flora. This treatment method is used to treat MDD disorders (4, 17, 18). New hypotheses regarding the development of depression have emerged, including the changes in the hypothalamus-pituitary-adrenal (HPA) axis, which is associated with the stress response, as well as the dysfunction in neurogenesis and neuroplasticity, which involves brain-derived neurotrophic factors (*BDNF*) (19).

Several different factors may contribute to depression such as stress, poor nutrition, insufficient exercise, obesity, chronic low-grade inflammation, smoking, atopic diseases, insufficient sleep, poor cleanliness, problems with dental care, and nutritional deficiencies, especially vitamin D insufficiency (20, 21). The study of the therapeutic effects of gut microbes on behavioral and cognitive function will lead to innovations in depression treatment (22). The human microbiome is now well understood by researchers because of significant advances in sequencing technology. This knowledge found the significance of symbiotic microbes in the body, particularly the gut microbiome (GM), for maintaining homeostasis (23, 24). This may contain 10–100 trillion symbiotic microbes, including bacteria, viruses, fungi, protozoans, and archaeal (25), most of these are intestinal bacteria, which belong to the phyla *Firmicutes* and *Bacteroidetes* (26, 27). Gut bacteria play a crucial role in maintaining host health through essential metabolic processes, supporting, and developing the immune system, and providing defense against pathogen invasions (28–30). Numerous neurological and psychiatric disorders, including conditions such as autism spectrum disorder, anxiety, and depression, have been associated with dysbiosis.

**Figure 2:**
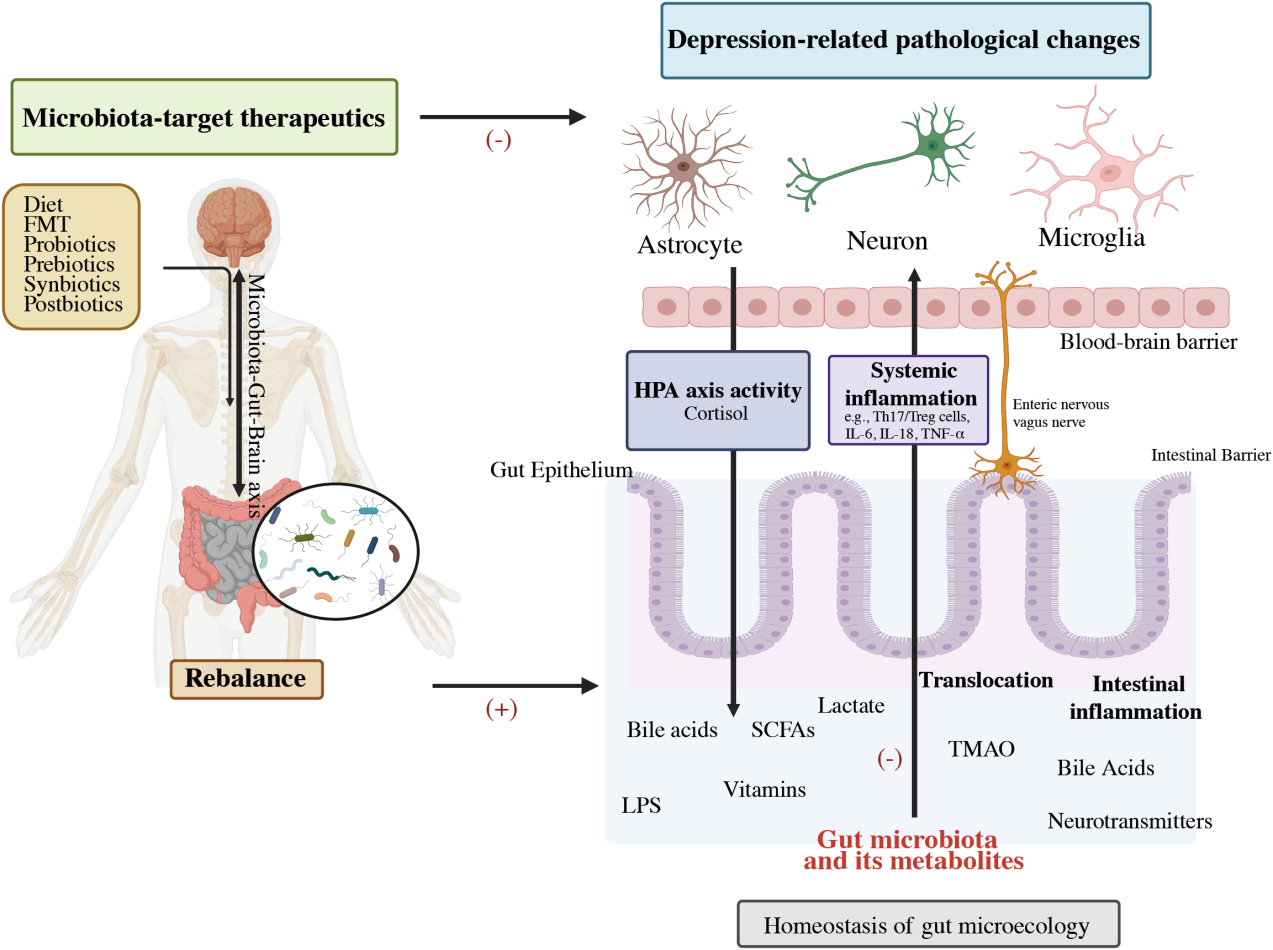
Potential role of the gut-brain-microbiota axis in depression pathophysiology and its therapeutics. FMT, Fecal microbiota transplantation; HPA, Hypothalamic–pituitary–adrenal; SCFAs, Short-chain fatty acids; LPS, Lipopolysaccharide; TMAO, Trimethylamine-N-oxide; IL-6, Interleukin-6; IL-1β, Interleukin-1β; TNF-α, Tumor necrosis factor-alpha (32).

The Microbiota-Gut-Brain Axis (MGBA) theory has advanced the most current depression hypothesis. (2, 31). The contribution of the MGB axis avenues in depression has become well-established as shown in **Figure 2** (32). Depression-related physiological dysbiosis is thought to involve deficient metabolites and neural networks, lower growth of neurons, reduced neural plasticity, and brain inflammation (33–36). Current research indicates that the MGBA exerts either a direct or an indirect influence on MDD via the gut microbiota. This concept acts as a significant impetus for discovering mechanistic studies and exploring microbial biomarkers within the realm of depression (4, 37, 38). Prebiotics facilitate the proliferation of healthy probiotic bacteria and enhance the gut-brain link, have the potential to increase the influence of probiotics on the adult neurometabolism (39). Recent studies suggest that post-biotics might also influence the activity of the Brain-Gut-Microbiota (BGM) axis (40). This review aims to offer a comprehensive perspective on the interplay between colonic microbiota and depressive disorders, with a specific focus on elucidating the association of intestinal microbial relation with depression progression. Moreover, it explores the therapeutic possibilities of probiotics, prebiotics, and related factors in addressing depression.

## 2 Microbiota-gut-brain axis and depression

**Figure 3:**
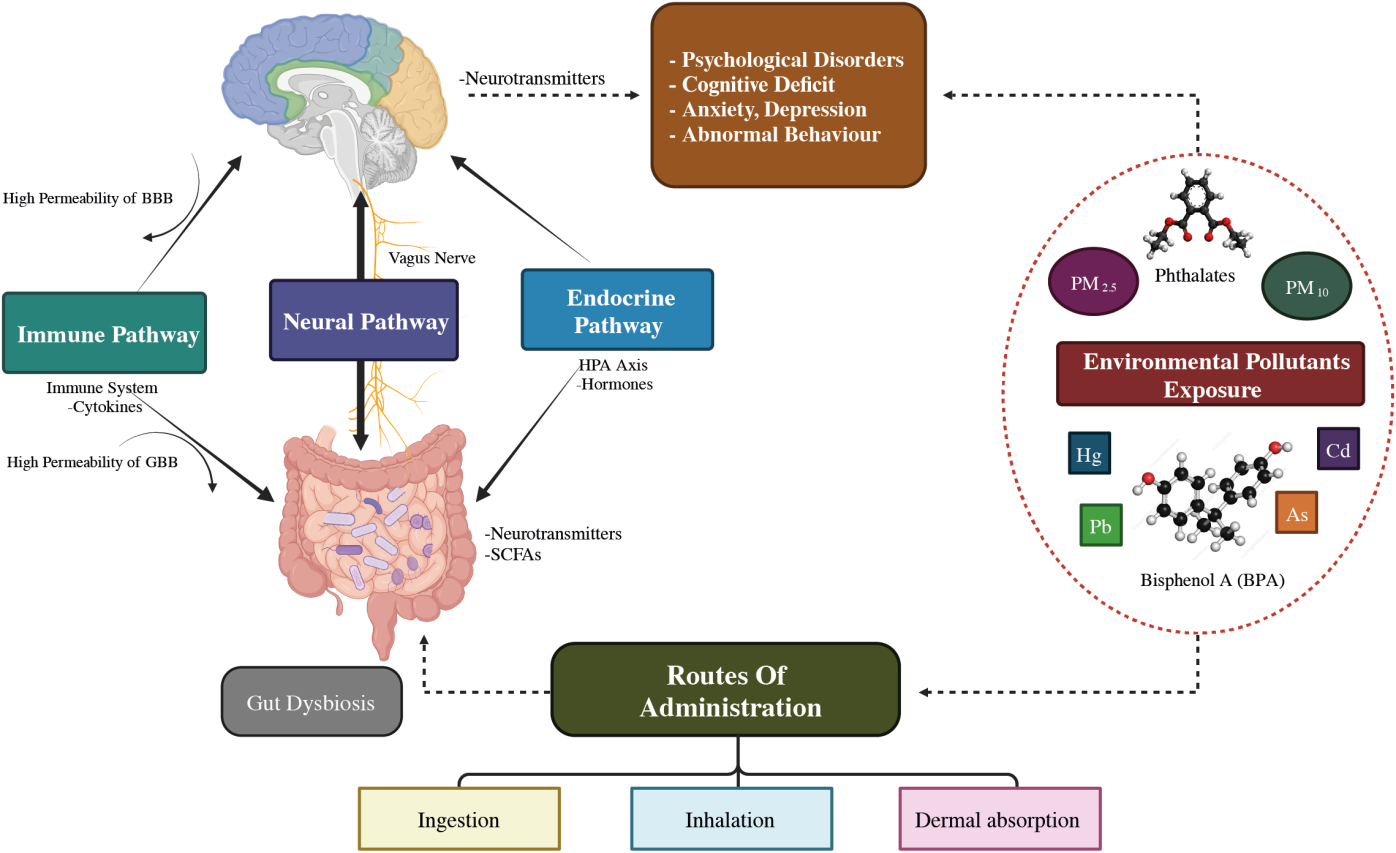
The illustrative demonstration of possible bidirectional linkages that regulate the microbiota-gut-brain (MGB) axis. BBB, Blood–Brain barrier; GBB, Gut–Blood barrier; HPA axis, Hypothalamus–Pituitary–Adrenal axis; SCFAs, Short chain fatty acids; Cd, Cadmium; As, Arsenic; O3, Ozone; BPA, Bisphenol A; PM, Particulate matter; ↑: increased/higher (53).

The MGB axis concept has arisen in the psychiatric field, as to how microbiome contributes to human homeostasis (41). The microbiota-gut-brain axis shows an association between the brain and the billions of bacteria in the intestinal tract (42). The human GI tract contains up to 10^14^ microbe cells, which comprise the microbiota (43), which is an umbrella term for all microorganisms living on or in the human body (44). About 75% of the microbiome is made up of the two main taxa *Bacteroidetes* and *Firmicutes* (45), and *Proteobacteria, Actinobacteria, Fusobacteria*, and *Verrucomicrobia* are found in comparatively small amounts (46). Recent investigations have revealed the digestive microbial tract has an enormous impact on human brain processes through the gut-brain axis (47). In particular, MDD patients have an increased *Bacteroidetes*/*Firmicutes* ratio, which is considered an improvement of the genus *Bacteroides* and a depletion of the genus *Blautia*, *Faecalibacterium*, and *Coprococcus* (48–50). Further, *Eggerthella* levels were consistently higher and *Sutterella* levels were consistently lower in people with MDD (49, 51). The ability of the microbiota to affect CNS activities bidirectionally through the vagus nervous system (VNS) is mainly mediated by the neurological (ANS), hormonal (HPA axis), and immunological (cytokine and chemokine) processes, all of which are interrelated (52). Exposure to biological contaminants may promote intestinal dysbiosis, which disrupts the GBA’s regular activity and contributes to psychological conditions such as anxiety, stress, depression, and mental disorders as illustrated in **Figure 3** (54).

Moreover, increased systematic translocation of intestinal metabolites, bacterial particulates, or even microbiota through the damaged intestinal tract (the “leaky gut”) heightens systemic inflammatory reactions such as Th17/Treg disorder, interleukin-6, IL-1, and tumor necrosis factors alpha that are being implicated in the etiology of depression. The enteric nervous system (ENS), also known as the “second brain,” is linked to the formation of brain disorders (55). By affecting gut secretion, immune defenses, motility, and permeability, aberrant ENS action caused by intestinal disorders exacerbates depression-related clinical modifications. The stimulation of the vagus nerve was initially approved in clinical studies for treatment-resistant depression. Bacterial cells, such as lipopolysaccharides (LPS) and peripheral inflammatory mediators enter the brain via BBB, causing neural inflammation, and subsequently induce neuropathological changes, such as synaptic defects, degeneration, abnormal neurons, and brain chemicals discharge which contribute to the development of depressive disorders. Excess cortisol promotes gut disease as part of the brain-gut axis by altering the integrity of intestinal barriers and inflammatory responses, leading to a leaky gut; this pathway is an important aspect of the MGB axis in depression. Other signal transduction systems and metabolic pathways involved in the MGB-based cause of depression include the endocannabinoid system, CAMKII-CREB and MAPK signaling, and glycerophospholipid metabolism. These bidirectional gut-brain communication routes form a complex network of systems, and their interconnections confounded studies into the mechanisms behind the microbial modulation of depressive disease. Ultimately, the MGB axis is responsible for the systemic diffusion of several hormones and metabolites into the bloodstream (56–59).

## 3 Composition of gut bacteria and the GBA

**Figure 4:**
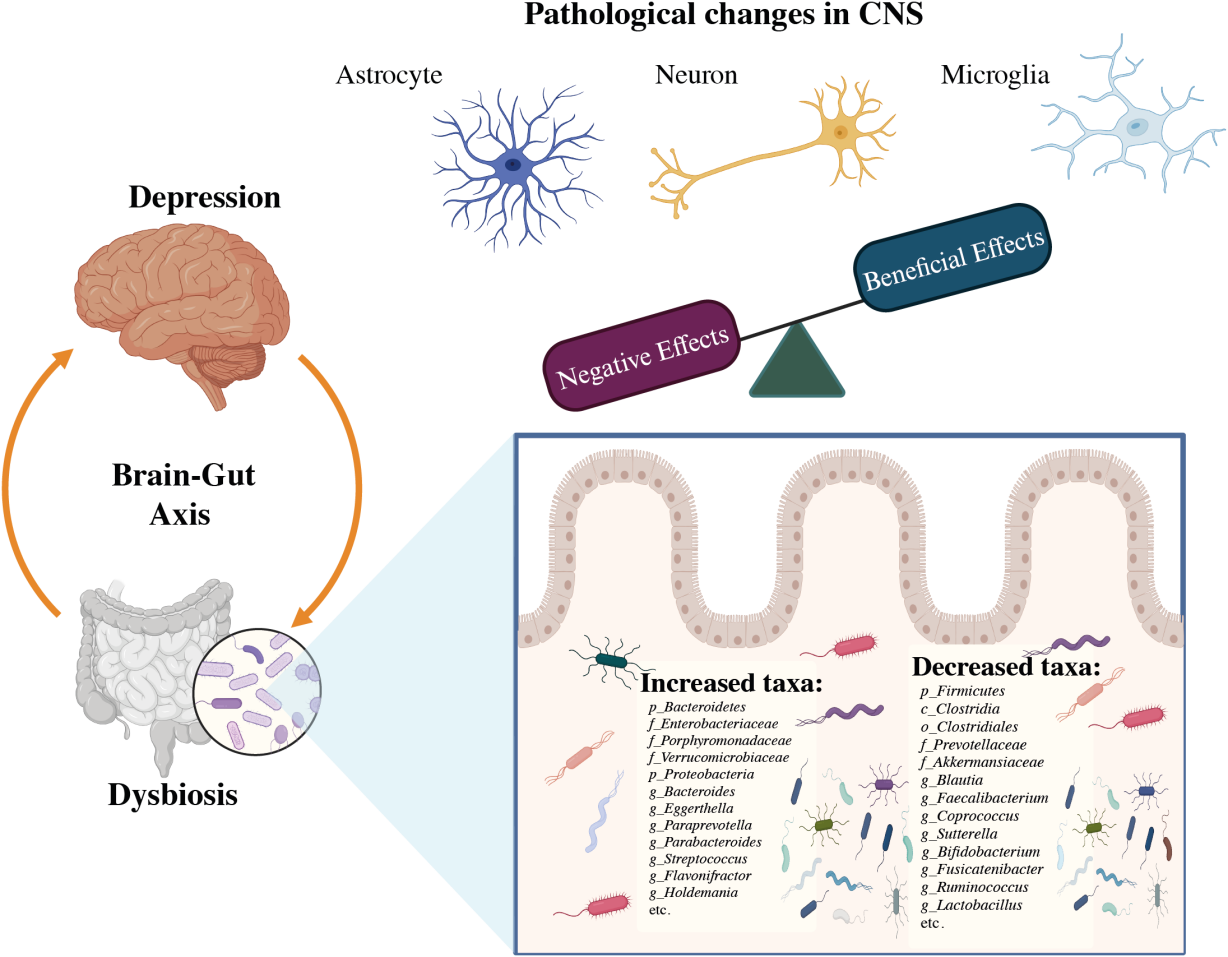
The association between dysbiosis and brain infectious modifications during the course of depression (32).

The composition of gut microbial communities varies among individuals, and approximately 70% of species-level phylogenetic types are distinct to each person (60). Several studies have found a link between dysbiosis and depression by comparing alterations in the gut microbial profile of sufferers of MDD to healthy persons, notably in terms of the variety of bacteria and relative number of specific microbial taxa as shown in **Figure 4**. In Depression, *Firmicutes* (primarily the genera *Clostridium, Enterococcus, Lactobacillus*, and *Faecalibacterium*) and *Bacteroidetes* (particularly the species *Bacteroides* and *Prevotella*) account for over 90% of the adult gut microbiota (61). *Proteobacteria, Actinobacteria, Fusobacteria*, and *Verrucomicrobia* are some of the other phyla found in MDD patients. In MDD patients with MDD, there was a rise in *Eggerthella* and a decrease in *Sutterella* (62). The initial phases of psychosis appear to be caused by a dysregulated fungal-bacteria interaction, and gut fungal dysbiosis has been associated with depression (63). The microbiota also includes eukaryotes such as yeasts and protists, viruses, and a few archaea species (*Methanobrevibactersmithii*) (64, 65). Studies on animals have shown that a gut microbe imbalance alters neurological chemistry, metabolic status, and brain activity (66). By overexpressing tight junction proteins and maintaining the blood-brain barrier (BBB) integrity, short-chain fatty acids (SCFA) can penetrate the BBB via monocarboxylate transporters (MCTs) (67). The SCFAs acetate (C2), propionate (C3), and butyrate (C4), produced by various microbial species in the gut are the most prevalent anions or metabolites in the gut which have a substantial role in psychiatric diseases (68, 69), in both direct and indirect ways (67). Modifications in neurotransmitter levels by modulatory pathways such as the kynurenine pathway, and also changes in accessibility and effects of SCFAs in the brain, may have an impact on *BDNF* activities such as neural survival and CNS development (70).

**Figure 5:**
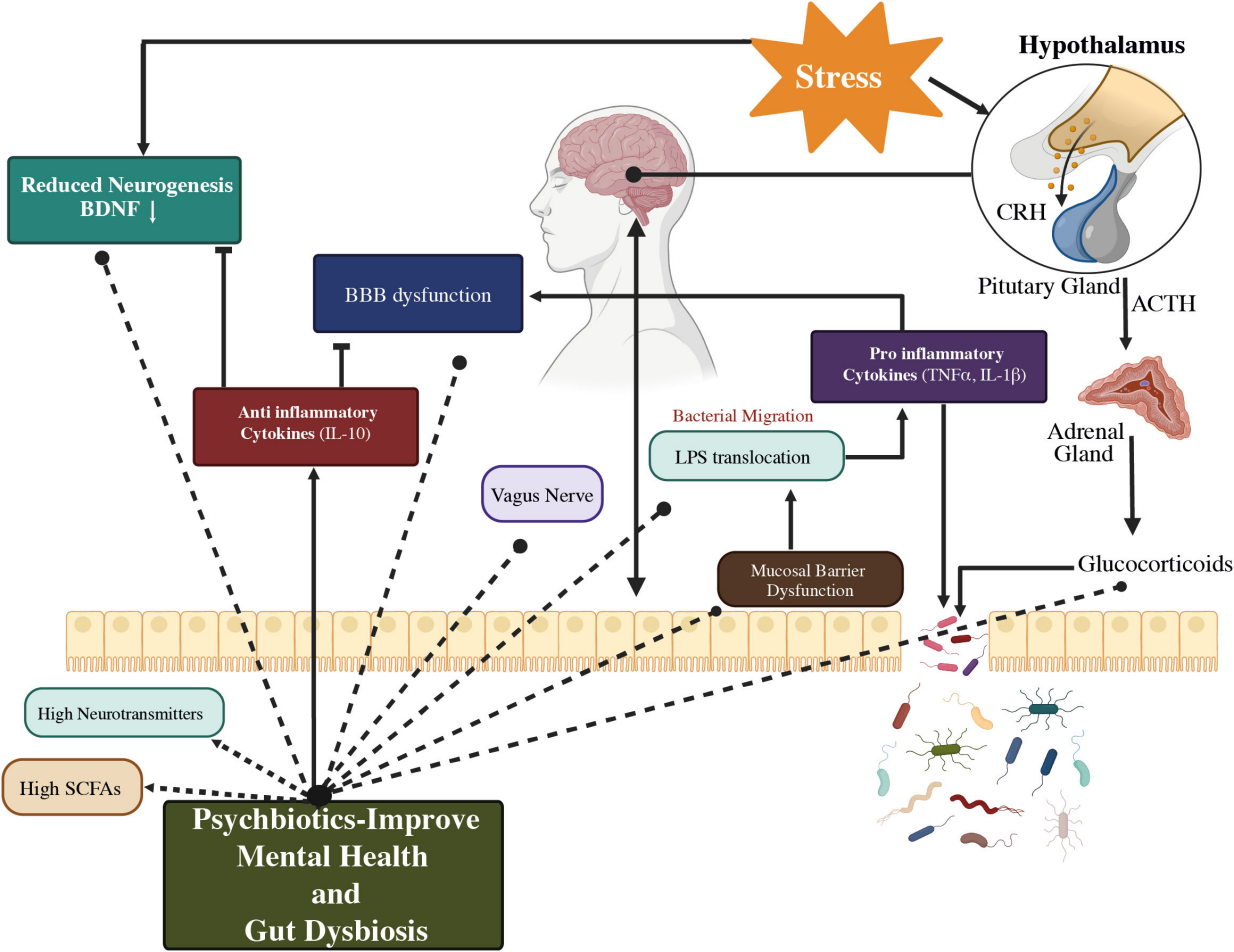
A representation of a proposed method of action for psychobiotics, involving intestinal microbiota modification. Psychobiotics treat psychological conditions by lowering inflammation, repairing gut permeability, restoring BBB integrity, modifying neurotransmitters, regulating the HPA axis, and increasing SCFA levels. BBB, Blood–Brain barrier; HPA axis, Hypothalamus–Pituitary–Adrenal axis; CRH, Corticotrophin-releasing hormone; ACTH, Adrenocorticotropic hormone; SCFAs, Short-chain fatty acids; IL-10, Interleukin-10; TNF α, Tumor necrosis factor α; BDNF, Brain-derived neurotrophic factor; LPS, Lipopolysaccharides; ↑: higher/increased (53).

The SCFAs also influence the secretion of intestinal hormones such as peptide tyrosine (PYY), cholecystokinin (CCK), and glucagon-like peptide-1 (GLP-1) from gut mucosal enteroendocrine cells that express free fatty асid receptors (FFARs) (71, 72). Blood-borne PYY and GLP;-1 enter the cerebellum of rodents and have major effects on neurotransmitters and behavior. Daily exposure to a wide range of environmental pollutants alters our gut microbes, inhibits bidirectional GBA, and may result in the development of psychiatric diseases (53). Even so, it was demonstrated that the primary mechanisms by which psychobiotics exert their effects include HPA axis regulation, immune response and inflammation modulation, and neurohormone and neurotransmitter synthesis as depicted in **Figure 5** (70).

The pathophysiology of various mental diseases, including anxiety and MDD, is linked to gut microbiota dysbiosis (73, 74). It was shown that some pathogens and enterotypes have significant contributors to induce mental disorders. A total of six microbial taxa were found to have adverse interactions, whereas *Hungatella* and *Fusicatenibacter* showed favorable associations, with *Butyricicoccus, Clostridium, Desulfovibrio piger, Parabacteroides merdae*, and *Hungatella* showing positive correlations, with the depressive phenome. Patients with mood disorders consistently had lower levels of *Faecalibacterium* and greater levels of *Actinobacteria* and *Enterobacteriaceae* (75). A second enterotype found a decrease in high-density lipoprotein cholesterol (HDLc) and an increase in the plasma atherogenic index (76), and the results reveal that certain microbes are possibly associated with psychological disorders and their manifestation (77). Potential advances in treatments and medical biomarkers may result from an improved comprehension of the functions played by different enterotypes and pathogens in mental health issues (51).

**Figure 6:**
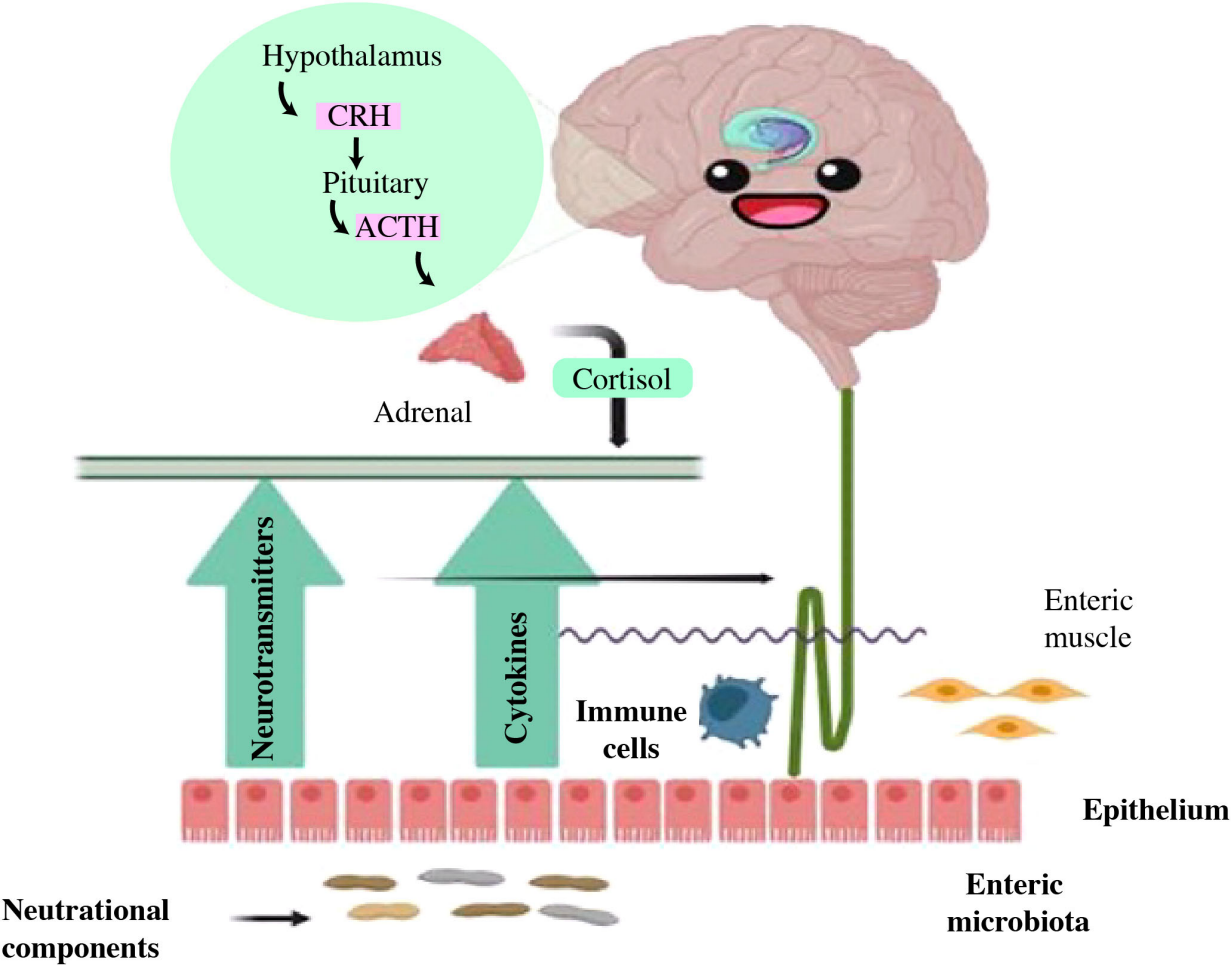
The reciprocal interaction between gut microbiota and the brain. Gut microbiota may modulate brain function and development through immune signaling (anti-inflammatory cytokines, chemokines, and immune cells), endocrine, and neural pathways. The brain may influence the gut through neurotransmitters that impact immune function and through alterations in cortisol levels, intestinal motility, and permeability. Nutritional components may exert effects on each of these communication pathways (86).

Moreover, several risk factors were linked to the pathophysiology of intestinal dysbiosis, antibiotic usage was shown to both transiently and permanently alter the composition of the gut microbiome (78, 79). Obesity, high-fat diets, and high-sugar diets have all been associated with reproducible gut microbiome modifications (80–82). It is also thought that exposure to environmental pollutants have a role in the development of gut dysbiosis at different stages of life (83, 84). In addition, related to gut dysbiosis include xenobiotics, such as exposure to pesticides and heavy metals, as well as social stressors (50, 85). The interaction is facilitated by the microbiota, but a dysbiotic biota can lead to brain inflammation and psychopathologies as depicted in **Figure 6** (86, 87).

Both clinical and preclinical research has found the influence of bacterial alterations on depression concerning inflammatory bowel diseases (IBD). Over 20 percent of individuals with IBD experience depression and difficulty sleeping. Interventions targeted at reducing inflammation have become increasingly popular among both IBD patients and healthy individuals since inflammation impacts brain tissue and their cognitive abilities (88). Individual’s distinct intestinal bacteria are determined by their surroundings, developmental stage, and genetic profile (89). The etiology of depression is often linked to the intestinal MGB axis, and it overlaps with the pathways that contribute to the progress of IBD and obesity (90). Studies conducted on individuals exhibiting depression symptoms showed that using probiotic supplements may have minor antidepressant properties (91). The gut’s reciprocal association with the neural network affects the formation of local inflammatory damage and psychophysiological sensitivity in IBD individuals (92). Depressive disorders and increased stress sensitivity are often associated with IBD, which might impact the progress and severity of the disorder. In general, the gut-mood correlations in this instance of IBD have been confirmed by the medical investigations (93).

The gut microbiota has evolved more pathogenically over time due to succession and within-host evolution, which may have caused unique microbial traits in MDD patients of different ages (48, 94, 95). Many studies explored age and gender as treatment variables for depression. Globally, women are prone than men to experience depression-related disorders (96). The probability of mood disorders decreases with age, and the difference in frequency between young men and women vanishes in individuals over 75. It was found that the hyperlink between depression and married life varies with age and gender. The risk rates for depression were lower for widowed, single, or divorced women compared to married individuals (97). In 2019, 4.2% of individuals identified with moderate symptoms, 11.5% had mild symptoms, and 2.8% of adults reported having profound signs of depression. The demographic of 18 to 29 years old comprised the highest number of adults with signs of depression (21.0%), followed by 45 to 64 years old (18.4%), 65 and older (18.4%), and 30-44 years old (16.8%). Women were more likely than men to experience moderate, severe, or mild manifestations of depression (98). Studies found an association between markers of quality of life (QoL) and certain intestinal microbes (*Faecalibacterium* and *Coprococcus*) associated with depression (99, 100).

The term “quality of life” encompasses a broad range of elements, including living conditions, opinions, social contacts, mental health, and degree of independence. It was used as a predictor of both the efficacy of therapy and depression relapse. The QoL level is similarly lower in euthymic depressed people, and this can be impacted by mental abilities, clinical symptoms, and social factors. A few studies have looked at the QOL of euthymic individuals with MDD and depression (101). After considering antidepressant effects, it was discovered that depression correlates with a depletion of the number of *Dialister* and *Coprococcus* spp. (102). Moreover, bacterial production of the dopamine metabolite (3,4-dihydroxyphenylacetic acid) is strongly linked with mental QoL, while γ-aminobutyric acid is associated with clinical depression. They provide evidence suggesting the intestinal microbes and QoL variables have mental health implications (93). Furthermore, animal studies demonstrate that the microbiota in the gut can alter the neurological aspects of depression (103), such as low-grade immune activation (104), the HPA activity (105), altered tryptophan metabolism (106), and neurotrophic factors (107, 108).

Some research indicates that transplanting the microbiome from one animal (either stressed or fat) to another control animal can significantly alter anxiety-like behaviors, which are a common comorbidity of depression (109). Different *lactobacillus* and *bifidobacterial* species have been found in animal models to influence depression and stress-related behavior (110–112). Recently, an abundance of experimental research was conducted, especially on animals, to discover how the gut microbiota influences GBA (27). Prebiotic and probiotic use may benefit mood and anxiety aspects, alter HPA function, and alter brain activity, according to a growing body of research conducted on healthy individuals (113, 114).

Moreover, bacteriophages help the microbiota of bacterial species perform their functions by increasing the range of the colon microbial alleles and traits (115). It demonstrates that MDD patients have changed the bacteriophage population, particularly in the phylum *Caudovirales* (48). Prior reviews covered the part *Candida albicans* plays in the pathogenesis of psychological syndromes (116), owing to the intricacy of interactions between bacterial and nonbacterial gut microbiota. Antibiotic usage, persistent stress, and a poor diet are other factors that impact gut flora and raise the risk of depression (117, 118). Multiple studies are being conducted on the intestinal microbes of developed countries; however, there is a dearth of research on indigenous tribes that are not subjected to similar stresses and situations as modern developed nations. Recent studies have highlighted the potential link between microbiota and mental disorders in such groups. It was revealed that MDD individuals have altered β, and α-diversity as well as a reduction in butyrate-producing bacteria. Initial studies suggest that the microbiome may be utilized to predict treatment outcomes and diagnose psychological problems. Moreover, clinical study outcomes support the use of probiotics as an adjunct in the MDD treatment, and determine the efficacy of psychobiotic therapy in aboriginal tribes with psychiatric conditions (119, 120). However, the induction of the gut bacteria via prebiotics, probiotics, or antibiotics, and specific FMT-based studies, is an effective therapeutic or preventative measure to counteract behavioral and cognitive problems, and these may be useful to supplement the action of drugs for the management of brain disorders (121).

### 3.1 Antibiotic use in neurological disorders and potential side effects

The first known case of antibiotic-induced depression was reported in 2010, and it may be a contributing factor to anxiety and depression. A well-known antibiotic minocycline has demonstrated promising results in treating depression in both humans and animals (122–124). The monoamine theory of depression led to the development of both tricyclic antidepressants (TCAs) and serotonin-specific reuptake inhibitors (SSRIs), which both increase central monoamine levels. This concept is still being studied in great detail. Several newer antidepressants, such as the SSRI, and fluoxetine have antibacterial activity to treat depression (125, 126). The improper use of antibiotics can significantly affect the diversity of bacterial species, potentially leading to both short and long-term shifts in the composition of intestinal bacterial flora. This can subsequently result in dysbiosis and disruptions in intestinal function (69). The field of administration of antibiotics is a significant issue, as seen in **Table 1** (127–129). Based on a detrimental clinical study, the administration of beta-lactam intravenously, comprising ampicillin, sulbactam, and cefazolin, not only disrupts the balance of bacteria but also affects the synthesis of powerful metabolites. Horizontal gene transfer (HGT) and antibiotic resistance genes both exist. Since probiotics, commensal, and opportunistic infections can survive against antimicrobial drugs by passing resistance genes, this has several therapeutic consequences. Although gene therapy and personalized drugs could treat antimicrobial resistance, probiotic-induced resistance requires significant interventional study to uncover the host-specific antibiotic-resistant genes (130). Antibiotics can alter the gut microbiota, and microbes can also modify the drugs, altering their chemistry and leading to the development of drug resistance.

**Table 1:** **A** list of the numerous antibiotics that are commonly used against depression.

Antibiotic	Class of antibiotic	Broad/narrow spectrum	Mode of action	Target pathogen
Amoxicillin, ampicillin	β‐Lactam	Broad‐spectrum	Bactericidal	Rhinosinusitis, respiratory and genitourinary tract infections, septicemia
Cephalosporin	β‐Lactam	Broad‐spectrum	Bactericidal	Urinary and respiratory tract infection, Gram‐negative bacteria
Azithromycin/erythromycin	Macrolides	Broad‐spectrum	Bactericidal	Sinusitis, pneumonia, respiratory tract, and skin infection, urogenital and chlamydial infection
Metronidazole	Nitroimidazole	Broad‐spectrum	Bactericidal	Protozoal infections, *C. difficile* infections, Gram‐negative bacterial infections
Gentamycin	Aminoglycoside	Broad‐spectrum	Bactericidal	Urinary tract infection, Gram‐negative bacterial infections

### 3.2 Chronic stress

The term “Stress” identified by Hans Selye, defined it as the body’s entire reaction to demands placed on it. He identified the three different stages of the body’s stress response including alarm, resistance, and weariness. He also separated stress into two types such as acute (short-term) and chronic (long-term), depending on how long the stressful event lasted (131, 132). Stress is a prevalent risk factor for over 75% of both physical and mental health conditions, contributing to elevated morbidity and mortality rates (133). Chronic mild stress (CMS) is widely cited in the literature as a substantial predictor of having a severe depressive episode (1, 134). Historically, the attention was mostly on traditional pathways, the HPA axis, and the sympathetic nervous system that link stress to disease. Inflammation has come to be a key mechanism behind the adverse consequences of anxiety (135–139). Depressed has also been associated with disruptions in the immune system, particularly the reduction of natural killer cell activity. Furthermore, preclinical models indicate a link between depression and prolonged stress. In such models, persistent corticosterone (CORT) treatment, exposure to erratic moderate stressors, and social defeat are standard techniques for producing depressive-like behaviors in animals. These actions comprise feeling helpless and anhedonia, two hallmarks of depression symptoms (140, 141). Interleukin (IL)-1b and TNF-alpha are two inflammatory markers that have significantly risen in animal models as a result of increased stress hormone production (142). Individuals suspected of depression usually have higher serum or plasma concentrations of several cytokines, including TNF-α and its soluble receptor TNFR2. This data is derived from a meta-analytic investigation that concentrated on the main cytokines examined. Conversely, individuals with depression appear to have lower IFN-α levels than healthy controls. These findings highlight the intricate link between the immune system and depression, suggesting that inflammation may play a role in the development of depressive symptoms (143–147). The higher levels of C-reactive protein (CRP), a widely employed indicator of inflammation, were found to be predictive of the onset of depressive symptoms (148, 149). More study is required to fully comprehend the potential link between depression and inflammation. Remarkably, a few studies indicate that inflammation may not be high in the early stages of MDD, which may indicate a poor prognosis for the first antidepressant medication. The differences in the duration or chronicity of the MDD may account for these disparities in inflammatory markers (150, 151).

### 3.3 Poor diet

Nutritional psychiatry is an emerging field of science that focuses on the relationship between dietary and mental health, brain function, and mental disorders (152). Understanding how diet and nutrition affect these pathways may provide important new information on effective MDD treatment strategies. One particularly effective method that has shown promise is the Mediterranean diet (MD), which may help improve the clinical outcomes for depressed patients (153). A study suggests that eating habits and dietary quality may affect the mental health of adolescents (154). Numerous studies have shown that poorer quality diets, such as the Western diet, can lower *Lactobacillus* levels, reduce the richness of the entire microbial community, and be associated with depression risk susceptibility (155). Twelve epidemiological studies were conducted to examine any possible links between trends in children’s and adults’ mental health and the quality of their diet (154). The intake of folate, zinc, and magnesium appears to be negatively connected with depressive disease, which means that a larger intake of these nutrients is linked to a decreased risk of depression. Because of the opposite link between omega-3 fatty acids and anxiety disorders, a higher intake of omega-3 fatty acids corresponds to a decreased risk of developing anxiety disorders (156). Therefore, dietary interventions are emerging as highly promising avenues of exploration in the realm of MDD. It is attributed to the presence of certain nutrients such as omega-3 fats, vitamins, polyphenols, and cocaine, as well as foodstuffs such as fish, nuts, seeds, vegetables, fruits, coffee/tea, and fermented goods, which are currently being studied. As demonstrated in **Table 2**, dietary supplements such as S-Adenosylmethionine, acetylcarnitine, creatine, and amino acids have been investigated (157, 158). The study of eating habits and the broader nutritional understanding is still a potential field of investigation. Interventions that use the Greek diet show substantial advantages to those suffering from profound depression. However, further research is needed to confirm their value and completely understand the multifaceted relationship between nutrition and the MDD (159, 160).

**Table 2:** **Main nutrients and dietary recommendations** with potential antidepressant effects.

Nutrients	Food	Antidepressant Mechanisms	Studies in MDD
**Omega 3 PUFA**	Fish and seafood (DHA and EPA), seeds and nuts (ALA)	Epigenetic modulation; Anti-inflammatory; Prebiotic; Increase in membrane fluidity; serotonin transport; enhanced dopamine concentration and dopamine 2 receptor in the frontal cortex; cellular signaling.	**DHA and EPA** either by supplementation or contained in a high intake of fish (≥2 times per week exerted relevant protective and antidepressant effects.
			**ALA** contained in seeds and nuts (especially walnuts) can ameliorate depressive symptoms and prevent their onset.
**Vitamin D**	Fish and animal products; mushrooms; fortified products	Vitamin D influences the immune and gut microbiota modulation; Serotonin synthesis; Circadian clock regulation and augmented BDNF production, exerting synergic effects with omega 3 PUFA.	Controversial results have been obtained regarding the possible antidepressant effect of **vitamin D** intake; although, its low consumption worldwide can be involved in the high prevalence of MDD.
**Vitamins from the B complex**	Fruits and vegetables/Animal products or supplementation (B12)	Anti-inflammatory and pleiotropic actions.	Low levels of **B1**, **B2**, **B3**, **B6**, **B9**, and **B12** vitamins can be related to a proinflammatory status of the immune system.
			Some studies have found slight benefits from supplementing with B6 or B12 alone or integrated into the B complex. However, daily intake above 400 g of fruits or vegetables is the most adequate approach to elevate the serum levels of vitamins
**Other vitamins**	Fruits and vegetables, animal products	Antioxidant, anti-inflammatory, and pleiotropic effects	There is some preclinical evidence supporting the supplementation with **provitamin A and E** as antidepressants; **Vitamin C** has been explored as an adjuvant with fluoxetine in children but not in adults; High intake of **vitamin K** (>232 μg/day) had significantly lower odds of developing depressive symptoms at baseline and each per 100 μg/day the odds of this condition decreased by12%. Daily intake above 400 g of fruits or vegetables is the most adequate approach to elevate the serum levels of vitamins.
**(A, C, E, K)**			
**Dietary fiber**	Fruits and vegetables	Prebiotic and modulatory effects in the MGB axis; SCFA modulation.	1 g of fiber per 1000 kcal exerts protective actions of **fiber** against depression in premenopausal, but not postmenopausal women.
**Polyphenols**	Fruits and vegetables/Coffee and tea	Pleiotropic effects	1000 mg/day of **curcumin** but not 500 mg/day may be used as adjunctive therapy for patients with MDD Preclinical models have shown that doses between 10 and 80 mg/kg/day, of **resveratrol**, provide antidepressant effects; although, higher doses had the most significant benefits.
			**Quercetin** has provided a potential antidepressant role *in vivo*, ameliorating lipopolysaccharide (LPS)-induced depressive rats, leading to the upregulation of BDNF and other molecular markers.
			**Anthocyanins**, mainly found in berries, are being investigated in mice models as prominent antidepressants, upregulating monoamines and neurotrophic factors such as BDNF.
			**EGCG** has proven to have some critical antidepressant effects in preclinical models, leading to an augmented BDNF, serotonin, and reduced stress hormones.
			**Caffeic acid** also provides significant antidepressant effects *in vivo*, especially when combined with antidepressants.
			**Chlorogenic acid** found in green coffee appears to be a major inhibitor of the monoamine oxidase A (MAO-A), upregulating serotonin levels and providing antidepressant actions.
**Caffeine**	Coffee and tea	Neuroprotective; Epigenetic modulation of neurons, immune and glial cells; Targeting of the dopaminergic system through the non-selective adenosine antagonist action.	A nonlinear response between **caffeine** consumption and depression was found, showing their most benefits above 68 mg/day and below 509 mg/day.
**Psychobiotics**	Fermented foods and beverages (Yogurt, kefir, soy-derived products, kombucha, *Laminaria japonica*, etc.*)*	Modulatory effects on HPA and MGB axis; inflammation; neurotransmitter production (monoamines, GABA, glutamate, acetylcholine); BDNF levels; host metabolism.	3 weeks with daily consumption of **probiotic yogurt** improved the mood in people with poor mood and other significant benefits on mental health by positively modulating the HPA axis; Whole-fat yogurt, but not low fat, was related to a decreased risk of depression in women.
			**Kefir** and kefir peptides appear to be a promising antidepressant tested *in vivo*, displaying a major modulatory role in the MGB axis, influencing host behavior, serotonin synthesis, immune system, BDNF/TrkB signaling, and GABA levels
			High adherence to **soy-based fermented products** in traditional Japanese regimes is associated with lower rates of depressive symptoms, aiding in the clinical management of depression and cognitive impairment; Soy-based milk with Lactobacillus brevis FPA 3709 (1 × 106 CFU/mL) demonstrated antidepressant efficacy in SD rats similar to fluoxetine.
			**Kombucha** can attenuate LPS-mediated neuroinflammation and oxidative stress markers, but direct evidence of its antidepressant role is needed.

## 4 Gut microbiota-derived metabolites and depression

Bacterial compounds found in the gut can enter the bloodstream affect brain regions and can interact with nearby gut components like hormonal cells or neurons, which can subsequently send signals to the brain (161). Numerous bioactive chemicals derived from gut microbes have been shown through experiments to affect the brain through direct or indirect methods. These processes can exacerbate neurological disorders or neuropathology when they become imbalanced (162). Numerous microbial species that may produce neurotransmitters, signaling chemicals, or metabolize their precursors into various substances can be found in the human microbiome (163–165), during food fermentation and bacterially altered host molecules including bile acids (BAs) (166), tryptophan metabolites, and SCFAs with CNS effects are also produced by the gut bacteria. The metabolites from the gut microbiota are a double-edged sword in depression. (167).

### 4.1 Short-chain fatty acids

Small organic molecules referred to as SCFAs are readily engaged in the large intestine and produced through anaerobic fermentation, primarily in the cecum and colon, from dietary carbohydrates that are typically indigestible by our bodies (168). Although there are conflicting reports of how SCFAs affect behavior, they are believed to have a role in digestive, immunological, and molecular processes. Acetate, butyrate, and propionate have been demonstrated to lessen the symptoms of depression in rats (169). According to Kelly et al. (170), transplanting the fecal microbiota of MDD patients into sterile rats led to an increase in fecal SCFA levels when compared to controls and sodium butyrate improved depressed behavior in rats. Bacterial butyrate has been demonstrated to control the tyrosine hydroxylase gene’s expression, which in turn controls the production of dopamine, adrenaline, and noradrenaline by suggesting to have antidepressant properties (24, 171). The SCFAs were shown their treatment had antidepressant benefits by reducing the permeability and the HPA axis, particularly butyrate (168, 169). The MGBA has suggested that acetic, propionic, and caproic acids may have an impact on when depression symptoms appear. For example, acetate helps to defend against enteropathogenic infections and strengthens the intestinal barrier (172, 173). The isovaleric acid was found to have a causal link between signs of depression and blood cortisol levels and appears to inhibit neurotransmitter release in the synaptic cleft (174). Certainly, SCFAs have been observed to enhance the production of anorexigenic neuropeptides and alter the concentrations of neurotransmitters such as glutamine, glutamate, and GABA in the hypothalamus. The tryptophan 5-hydroxylase 1, crucial for the synthesis of serotonin (5-HT), and tyrosine hydroxylase, a rate-limiting enzyme in the production of dopamine, adrenaline, and noradrenaline, are both positively regulated by SCFAs (175, 176).

### 4.2 Bile acid

Bile acids are naturally occurring chemicals produced in the liver. In mice, bile acids can breach the BBB by activating the nuclear receptor farnesoid X receptor (FXR) in the ileum. This activation leads to the production of fibroblast growth factor 19 (FGF19) or its equivalent, fibroblast growth factor 15 (FGF15), which can suppress the HPA axis. This suppression of the HPA axis can have a protective effect against depression (71, 177, 178). It suggests that elevated BA levels can serve as a type of depression defense. *Turicibacterales, Turicibacteraceae*, and *Turicibacter* were associated with the increase in BAs production (32). At present, the precise mechanisms through which secondary bile acids influence the development of MDD remain unclear. Additionally, it is uncertain whether certain antidepressant medications, like paroxetine, a selective SSRI, have an impact on the gut microbiota. To gain a comprehensive understanding of the role of secondary BAs in MDD and the potential effects of antidepressant medications on the gut microbiota and BA synthesis, further research is necessary. This ongoing investigation is crucial for elucidating the complex interplay between these factors and their relevance to the management and treatment of MDD.

### 4.3 Trimethylamine N-oxide (TMAO)

Trimethylamine-N-oxide (TMAO), a tiny chemical compound produced by gut bacteria, has lately been linked to postoperative cognitive and neurodegenerative impairment (179). Foods containing phosphatidylcholine and L-carnitine are included in diets such as eggs, red meat, cheese, and sea salt fish. The gut microbiota converts these nutrients into trimethylamine (TMA), which is then oxidized by the hepatic FMO-1 and FMO-3 enzyme family members (180). Therefore, a variety of influencing variables, including diet, the gut microbiota, liver function, and the GBB, which controls the permeability of molecules produced from the gut into the bloodstream, affect serum TMAO levels (181, 182). Recently, it was demonstrated that variations in the TMAO concentration produced by gut bacteria are similarly associated with depression (183). The *Firmicutes* phylum contains the majority of the bacteria that produce TMA, including some *Actinobacteria* and *Proteobacteria* as well as *Clostridium* cluster XIVa and *Eubacterium* strains (184). Elevated production of TMA and its subsequent conversion to TMAO is associated with the potential to accumulate in various tissues, exerting effects on multiple organs and systems within the body. This phenomenon, notably affecting the cardiovascular system and the liver, demonstrates an adverse correlation with gut dysbiosis (185, 186). Prior research found an association between TMAO levels and the severity of depressive symptoms in both males and females. Overall, further study is needed to understand how the gut microbiota contributes to the neurocognitive advantages of TMA/TMAO in people with MDD. Other microbiota-derived metabolites linked to MDD pathophysiology include LPS, lactate, vitamins, aromatic amino acids, and tryptophan metabolites (187, 188).

## 5 Epigenetic-modulated inheritance and depression

The estimated familial risk of depression is approximately 35%, compared to the 65–70% hereditary rates for bipolar disorder and schizophrenia (189, 190). Recently, over 80 replicable loci linked to MDD were found by a genome-wide association study (GWAS), each of having a negligible impact (191, 192). Besides, only a very small portion of the overall inherited risk remains defined by the major depressive polygenic risk rating determined by these genetic locations (191). It was suggested that many gene variations that cause genetic risk are still unknown and other factors are strongly linked to the chance of developing MDD and other anxiety-related diseases, as shown by epidemiological research (193–195). It is becoming more widely recognized that variations in epigenetic modification lead to aberrant neuroendocrine responses, loss of neuroplasticity, neurotransmission, and dysfunction of neuroglia, all of which have been associated with the etiology of MDD and stresses such as anxieties and post-traumatic stress disorder (PTSD) (196, 197). Modifications in monoamine neurotransmitter function, altered stress-responsive mechanisms, and brain inflammation are all regulated by genetics. Moreover, epigenetic biomarkers have a role in the identification and treatment of MDD. Knowing the complex etiology and varied hallmarks of MDD may be enhanced by examining epigenetic changes, which could provide novel avenues for treatment (198, 199).

Although MDD supports their usage, the field of etiology research is still evolving by substituting peripheral epigenetic changes for brain activity. Nonetheless, it could be useful in developing biomarkers for disease and the efficacy of a treatment (200). Depression and anxiety may occur as a result of external variables such as parental stress, childhood trauma, and traumatic events in adults (201). Such variables may interact with genes through gene-by-environment interactions or direct epigenetic regulation. These processes include modifications to DNA, non-coding RNA, the structure of chromatin, histones, and so on (202). Several cytogenetic structural modifications associated with MDD are primarily unknown (203, 204). The m6A-methylated RNA has been identified by MeRIP-Seq methods offering a novel avenue for studying the transcriptome-wide expression of m6A in mental disorders. When combined with the widely used next-generation RNA sequencing methods, such research methods should enable an increased understanding of the possible pathways underlying depression (205).

There is evidence from new studies that brain chromatin receives metabolic signals (206). The regulation of glycolytic genes is regulated by lactate-dependent histone lactylation (H4K12a) in microglia, which results in microglia dysfunction and mental retardation (207). Further investigation is required to examine the role that metabolism-dependent genetic mechanisms play in the development of depression and whether these mechanisms can have effects similar to those of antidepressants. Moreover, several studies have revealed an association between genetics, the gut flora’s metabolites, and the intestinal microbiome, highlighting the influence of the microbiota on the synthesis of non-coding RNA (208, 209)., With the advent of novel mutagenic techniques such as Transcription Activator-Like Effector Nucleases (TALENs) and Clustered Regularly Interspaced Short Palindromic Repeats (CRISPR-Cas9), diseases related to the real induction of epigenetic loci at known stress-related DNA locations can be investigated (210, 211)., These advances allow us to focus on specific genes of the brain, and periods with epigenetic regulators to find out whether regulation of epigenetic changes at specific loci causes a distinct mental disorder (212).

## 6 The gut microbiota’s clinical application potential in MDD

**Figure 7:**
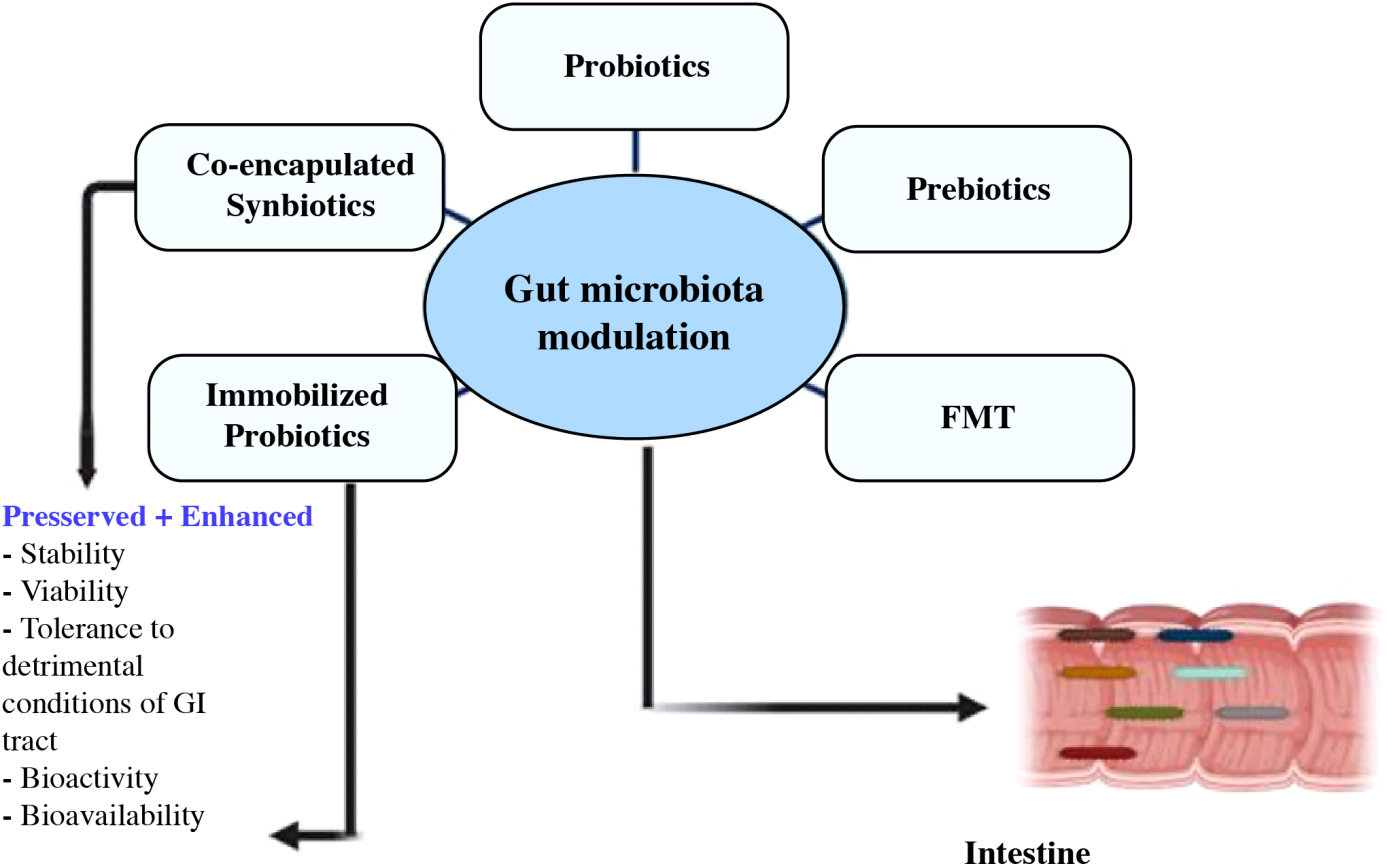
Utilizing modern techniques, co-encapsulated synbiotics and immobilized probiotics offer benefits in the targeted control of the gut flora (216).

By reducing neural inflammation and brain degeneration, probiotic, prebiotic, and fecal microbiota transplant administration may be a useful treatment for gut dysbiosis and enhance cognitive impairment (213, 214). To maintain a healthy balance, the activity of the gut bacteria can be controlled by diet, the use of drugs that encourage the growth of the microbiota, and the incorporation of microbes to nutrients (215). **Figure 7** depicts current approaches for modifying gut flora, with a focus on the health advantages offered by integrated synbiotics and immobile probiotics (216).

### 6.1 Prebiotic

The International Scientific Association for Probiotics and Prebiotics (ISAPP) has modified the definition of prebiotics to “a substrate that is selectively utilized by host microorganisms conferring a health benefit” and has expertise in microbiology, nutrition, and clinical research(217). The original prebiotic definition of chemicals based on carbohydrates was expanded to include non-carbohydrate substances such as PUFAs, polyphenols, etc. Prebiotics like inulin and short-chain carbohydrates work as a substrate for naturally occurring colonic bacteria like *Bifidobacterium* and *Lactobacillus*. Prebiotic use results in an increase in healthy bacteria in the gut microbiome, which benefits health (218).Prebiotics are classified as functional foods in the scientific literature due to their significance in enhancing health and avoiding infection (219). It includes the factors that promote the growth of beneficial microbes and the positive impact their products and metabolites have on health indexes. In addition to phenol, inulin, and chemicals from vegetables, herbs, and plants, fructooligosaccharides (FOS), galactooligosaccharides (GOS), and inulin are the most explored prebiotics and the prebiotics with the most evidence in the treatment of depression (220). The fructo-oligosaccharides and B-immunogalacto-oligosaccharides promoted the development of helpful bacteria like *B. longum* and decreased stress-induced activation of the HPA axis (221). However the anxiety and depressive-like behavior caused by LPS has been demonstrated to decrease after receiving B-immunogalacto-oligosaccharide (222). The prebiotics do not directly affect the body, but they do indirectly improve host health by encouraging the development of probiotics. As a result, prebiotics and probiotics are frequently used in the treatment of depression (223).

### 6.2 Probiotics

The name “probiotic” is derived from the Greek word “pro bios,” which means “for life,” and the positive impact of lactic acid fermentation products on human health has ancient origins (224). Both transient and native bacteria can be found in the human digestive system. The majority of native bacteria are a component of the commensal microbiota, even though some of them have been classified as potentially harmful or health-promoting. *Bifidobacterium* and *Lactobacillus* genera are the most common strains and potential sources of probiotics, and some of these organisms have powerful anti-inflammatory properties (225). Probiotics maintain the integrity of the gut barrier’s integrated function and reduce intestinal permeability, which lowers endotoxin levels (226, 227). Recent research suggests that the probiotic *lactobacillus rhamnosus*HN001 enhances psychological health in clinical populations (228). In 1910, Phillips’ study (229) published the first account in the literature of the beneficial benefits of probiotics in the treatment of depression. Miyaoka and colleagues examined the use of *Clostridium butyricum* (CBM588) as an additional therapy in patients with MDD who were resistant to treatment (230).The majority of probiotic regimens for the treatment of depression are based on *Lactobacillus* spp. and *Bifidobacterium* spp., according to a prior thorough investigation that found 178 species and subspecies of probiotics that can attenuate depression (231). Several probiotic species, including *L. helveticus*, *L. rhamnosus*, *B. longum*, and *B. breve* CCFM1025, are effective in treating clinical depression (**Table 3**) (232). A probiotic treatment dramatically raised the amount of *Lactobacillus* and reversed immunological alterations while reducing anxiety and depressive-like behaviors (233). Majeed and colleagues found a notable improvement in the symptoms of depression and IBS in patients treated with *Bacillus Coagulans* MTCC 5856 (234). The evidence suggests that *Lactobacillus plantarum* DR7 may offer benefits in terms of alleviating symptoms and improving psychological ratings. This particular strain of *Lactobacillus* is being investigated for its potential positive impact on various aspects of mental health and well-being (235).

**Table 3:** Effect of probiotics in depression and their significance.

Title	Disease	Probiotics effect	Significance
Antidepressants alter gut microbiota, and Ruminococcusflavefaciens can reverse their depressive-like effects	Depression	Ruminococcus, Adlercreutzia	Improved Depression
A randomized controlled experiment evaluating the clinical, gut microbial, and neurological advantages of probiotic add-on therapy in people suffering from depression.	MDD	Multi strain probiotic	Depressive symptoms have improved, and particular health-related bacterial taxa have been restored.
Probiotics and the Microbiota-Gut-Brain Axis: Focus on Psychiatry	Depression	Lactobacillus, Bifidobacteria	Improve mood by increasing good bacteria in the gut and stimulating the gut-brain axis.
Understanding the involvement of the microbiome in major depression	MDD	Lactobacillus spp.	The gut microbiome influences a range of mental diseases, including major depressive disorder (MDD).
Bacteroides species differentially modulate depression-like behavior via gut-brain metabolic signaling	MDD	Bacteroides fragilis, Bacteroides uniformis, Bacteroides caccae	Bacteroidetes contribute to depression vulnerability in mice through metabolic control along the gut-brain axis.
The gut microbiota and depressive symptoms across ethnic groups	Depression	Bifidobacterium genus	Ethnic variations in gut flora may explain some of the similar discrepancies in depression.
Identification of a Signaling Mechanism by Which the Microbiome Regulates Th17 Cell-Mediated Depressive-Like Behaviors in Mice	Depression	Lactobacillus farciminis	Bacteria alter mood.
The Role of the Gut Microbiota in the Development and Progression of Major Depressive and Bipolar Disorder	MDD	Bifidobacterium, Lactobacillus	The gut microbiota and anxiety, and also their relationship, to develop novel therapeutic strategies and lower the prevalence of treatment-resistant affective disease.
The role of Inflammation in Chronic Restraint Stress-Induced Depressive Behaviors: A Link Between Gut Dysbiosis and Neurotransmitter Disturbance	Depression	Lactobacillus, Ruminococcus, Oscillibacter	Inflammation has a role in microbiota dysbiosis and neurotransmitter metabolism disruption in CRS-induced depression.
Analysis of gut microbiota and intestinal integrity markers of inpatients with major depressive disorder	MDD	Paraprevotella, Clostridiales, Clostridia, Firmicutes, Alphaproteobacteria	Intestinal integrity and inflammatory indicators were linked to MDD patients’ responsiveness to therapy and symptom intensity.

### 6.3 Dietary interventions

Diet has been shown to have a significant effect on mood disorders and also on the composition and functionality of the gut microbiome (236, 237). There is an association between diet and depression, but because studies have been contradictory, the cause and effect of the association are still unclear. The Mediterranean diet (MD) (159, 160, 238), the Japanese diet (239), and the Norwegian diet (240) are just a few examples of “healthy” dietary patterns that are known for their abundance of vegetables, fruits, cereals, nuts, seeds, and pulses as well as their moderate amounts of dairy, eggs, and fish and unsaturated fats for treating depression. The MD diet which is abundant in nutritional polyphenols and fibers and has anti-inflammatory traits, is widely believed to be good for human wellness. Evidence shows that MD diet is linked to increased quality of life, a lower risk of developing metabolic disorders including diabetes and obesity, a lower risk of developing cancer and heart disease, and an improvement in mental health (241). The prevalence of depression is greater in those who follow a “Western” dietary pattern, which includes sugary and fatty meals, refined grains, fried and processed foods, red meat, high-fat dairy products, and low intake of fruits and vegetables (242, 243). The correlative investigations of healthy individuals revealed that eating “healthy” diets with lots of fruits and vegetables, moderate amounts of dairy, eggs, and fish, and unsaturated fats results in a decreased prevalence of depression (244). The association between depressive disorders and relative intake of carbohydrates is studied using DNA data from over 400,000 individuals. A 16% rise in calories derived from glucose was shown to be related to a 58% reduction in the rate of depression; the protective effect involves significant association (245).

Moreover, in France identified a link between bad nutrition and depression incidence, it was most likely due to reverse causation, with depression raising the likelihood of poor eating habits (246). Consuming meals high in sugar and saturated fats is prompted by depression symptoms, which cause gut dysbiosis and exacerbate depressive symptoms. It should come as no surprise that the Western diet, which is heavy in fat and sugar, raises intestinal permeability and pro-inflammatory signaling (247). However, a diet high in fiber, for instance, should be taken into consideration as adjuvant therapy for MDD since it stimulates the synthesis of immunomodulatory substances, such as SCFAs. In fact, *Bifidobacterium*, a species that was found in higher concentrations in the blood of MDD patients who consumed fermented milk products (248, 249). However, nutritional interventions should be used cautiously for the medical management of significant depressive disorders; they ought to be paired with other therapies such as behavioral therapy, medication for depression, and habitual modifications.

### 6.4 Synbiotics and postbiotics

A synergic combination of probiotics and prebiotics is referred to as a synbiotic, and prebiotics specifically have a role in promoting probiotic colonization of the gut (250). In a randomized experiment, the probiotic combination of *B. lactis*, *B. bifidum*, *L. acidophilus*, and *B. longum* with the synbiotics FOS, GOS, and inulin decreased depression and raised blood levels of a brain-derived neurotrophic factor in depressed patients compared to controls (251). According to this study, probiotics alone did not significantly reduce depressive symptoms as much as synbiotics did. Stated differently, despite the fact that the term “postbiotics” has been utilized in the literature for over two decades, there has only been a notable surge in research on its effects during the last five years (252). Postbiotics have therapeutic benefits that are comparable to those of probiotics in that they maintain the integrity of the epithelial barrier function, restore the variety and composition of the microbiota, manage immune reactions, and modulate signaling along the gut-brain axis (253). This presents a cutting-edge microbial treatment strategy for depression. According to studies, regulating the gut-brain axis protected mice against *Salmonella*-induced depressive-like behavior by pretreatment with heat-killed probiotic *L. plantarum*-derived Postbiotics, notably their metabolites (254). Some bacterial metabolites, including SCFAs and BAs have postbiotics properties. Sodium butyrate was administered orally to mice to cure depressive-like behaviors and to repair changes in gut barrier integrity and microbiota composition brought on by the drug paclitaxel(255). Similar to probiotics, Postbiotics may exert health benefits through similar mechanisms, such as improved epithelial barrier function, host-microbiota modulation, immune response modulation, systemic metabolism modulation, and nervous system signaling (256, 257).

### 6.5 Fecal microbiota transplantation

To balances the recipient’s unbalanced gut microbiota, FMT, which first emerged in China in the 4th century, entails injecting a healthy donor’s fecal solution into the recipient’s intestine (256). While only a few of the conditions that have been shown to benefit from FMT therapies include IBS, ulcerative colitis, and recurrent and refractory *Clostridium difficile* infection (258–260). Due to the postulated importance of the MGB axis in the pathophysiology of depression, the potential use of FMT as a therapeutic strategy to treat neuropsychiatric diseases (261). The GBA’s vagus nerve may be a crucial signaling mechanism that controls the antidepressant properties of FMT (170). Pre-clinical research has therefore demonstrated the contagiousness of gut microbiota-driven behaviors (64). FMT administration slowly decreased signs of depression in patients with diarrhea-predominant irritable bowel disorder, regardless of intestinal disease remission. These optimistic clinical outcomes were validated in individuals with concurrent IBS-D, stress, and depression by a randomized controlled study (RCT) (262). Despite the lack of research on FMT from healthy donors to depressed recipients, the potential for FMT to be beneficial in treating MDD stems from the transmissibility of behaviors linked to microbiota (263, 264). When developing medications related to FMT, it is crucial to maximize safety while considering both immediate and long-term adverse effects (265). In rodents, FMT-induced changes in the microbial makeup of the gut can modify behavior and have an impact on rodent models of psychiatric diseases (266). There have been no clinical trials on the use of FMT to treat depression. Similarly, FMT may provide antidepressant microbiota to depressed people, and anxiety is transmissible via FMT in mice (263). By using FMT, one may supplement harmful gut bacteria with healthy ones. So, it is possible to anticipate advantages in the immunological conditions used to treat neuropsychiatric disorders (267, 268). Even yet, more study is required before FMT may be used to treat depression. It’s fascinating to note that the clinical improvement caused by FMT was accompanied by an increase in the diversity of gut bacteria and a decrease in GI symptoms (269).

## 7 Conclusion, limitations, and future thrust

### 7.1 Conclusion

The maintenance of human health is significantly influenced by the gut microbiota, and research on the microbiome has advanced significantly, most notably in understanding how it functions biologically in depression. Examining whether gut microbiota and depression are related would allow researchers to address several significant concerns and potentially helpful treatments. To better understand the mechanisms of these disorders related to therapy, further research in the future is required on what causes the development of dysbiosis and depression, as well as drug-microbiota interactions. The gut flora and MDD have been associated in most clinical studies and experimental data about the microbiome’s involvement in the disorder. Research questions like “How does MGB signaling affect brain development?” have also been examined using stool samples. To develop a disease-based strain resource database, more research is required to identify the specific functions of the strains and elucidate the underlying mechanisms governing the gut-brain axis. This will need to identify the pathogenic and beneficial strains implicated in depression through the use of high-throughput sequencing, multi-omics approaches, and microbial culture technology. As adjuvant methods to boost coverage alongside the primary therapy, new probiotics (psychobiotics) or pharmabiotics containing bacteria that produce SCFAs and neuroactive metabolites are being researched. These bacteria inhibit normal depressive stress along the whole MGB axis. More studies and data, especially from randomized controlled trials, are needed to demonstrate the synergistic effects of integrative therapy such as diet and exercise on patients. Isotope monitoring, modified and CRISPR-edited strains, and other emerging methodological tools are assisting in the improvement of microbiota-based therapy methods for depression.

### 7.2 Limitations

An emerging body of evidence explores the role of intestinal microbiota in depression which presents challenging avenues where it is essential to admit various limitations inherent to this field of study. One of the illustrious challenges lies in constituting directionality and causality in the gut-brain axis, as the bidirectional exchange of information between these systems makes it complex to specifically determine whether the modulations in the microbiota composition are the main cause or consequence of depression disorder. Heterogeneity across the methods of different studies which include alterations in sample collection, data analysis, and sequencing techniques adds a further layer of complexity in drawing appropriate conclusions. Additionally, the diversity of microbiota and its sensitivity to an individual’s lifestyle, genetics, and geographical location further complexes the generalizability of observations. Available research studies are lacking in deep mechanistic insights which left the gaps in interpreting the molecular and cellular pathways interlinking the microbiome to depression symptoms. Clinical and preclinical trials demand microbiome-targeted strategies which should necessarily be based on rigorous, widespread, large-scale, and long-term experiments to further validate the safety and efficacy. Thus, while the role of the intestinal microbiome in depression disorder presents an entrancing exploration avenue, these limitations underline the necessity of moderate interpretation and comprehensive research.

### 7.3 Future thrust

The future interests of research into the complex association between the intestinal microbiome and depression promise a perspective shift in understanding and management of this intricate disorder. Overcoming current research limitations, future studies are braced to untangle the precise pathways governing the bidirectional exchange of information at the gut-brain axis. By rendering cutting-edge techniques that involve metagenomics and metabolomics, researchers can instantly identify particular microbial species and their linked metabolites which share critical roles in influencing neural mechanisms and behavior. Longitudinal probing can track microbiota dynamics before, during, and after depression events consequently will help in illuminating the causal relationship, potentially leading towards the determination of early diagnostic markers. Integration of a multitude of omics data such as genomics, microbiomics, and metabolomics, with individual clinical and preclinical profiling, will help in the development of tailored management strategies for individuals susceptible to microbiome-associated depression. Artificial intelligence (AI) tools and machine learning algorithms will enable the extraction of complex patterns from intricate datasets, disclosing novel correlations and directing the identification of potential targeted therapeutic measures. Incorporating research-based findings into effective intervention renders a potential frontier. Microbiome-targeted therapeutic interventions such as prebiotics, probiotics, and fecal microbiota can be promising as an adjunctive treatment for depression disorder. However robust preclinical and clinical trials are necessary to optimize these strategies and establish their efficacy and safety profiles. Moreover, investigations into the roles of diet in altering the gut-brain axis render a specific research avenue to promote mental health via nutritional strategies.

## Author contributions

AL: Writing – original draft. MH: Writing – review & editing, Supervision. AH: Writing – review & editing. MU: Methodology, Writing – review & editing. LZ: Writing – review & editing. MR: Writing – review & editing. AD: Writing – review & editing. KU: Writing – review & editing. WW: Writing – review & editing. GW: Funding acquisition, Supervision, Writing – review & editing.
